# Gonadal Disorder in the Thinlip Grey Mullet (*Liza ramada*, Risso 1827) as a Biomarker of Environmental Stress in Surface Waters

**DOI:** 10.3390/ijerph120201817

**Published:** 2015-02-05

**Authors:** Lorenzo Tancioni, Riccardo Caprioli, Ayad Hantoosh Dawood Al-Khafaji, Laura Mancini, Clara Boglione, Eleonora Ciccotti, Stefano Cataudella

**Affiliations:** 1 Laboratorio di Ecologia Sperimentale ed Acquacoltura, Dipartimento di Biologia, “Tor Vergata” University, Rome (RM), Via Cracovia 1 00134, Italy; E-Mails: tancioni@uniroma2.it (L.T.); boglione@uniroma2.it (C.B.); ciccotti@uniroma2.it (E.C.); cataudel@uniroma2.it (S.C.); 2 Istituto Zooprofilattico Sperimentale dell’Abruzzo e del Molise “G. Caporale”, Via Campo Boario, 64100 Teramo (TE), Italy; 3 Department of Biology, College of Science, Basrah University, Basrah 61004, Iraq; E-Mail: ayadayad1960@yhaoo.com; 4 Dipartimento di Ambiente e Connessa Prevenzione Primaria, National Institute of Health (ISS), Via Regina Elena 299, Roma (RM) 00181, Italy; E-Mail: laura.mancini@iss.it

**Keywords:** fish, mugilidae, gonadal disorders, bioindicators, water pollution, biomonitoring

## Abstract

The aim of this study was to evaluate the use of gonadal alterations in the thinlip grey mullet (*Liza ramada*) as a biological indicator in assessing aquatic ecosystems health, with particular emphasis to river ecosystems exposed to sewage discharges. For this purpose, the reproductive status and the presence of gonadal alterations were studied in 206 mullets collected from two sites on the low course of the Tiber River, downstream of a large urban sewage treatment plant and in the estuarine area, and from an uncontaminated pond considered as reference site. Intersex and irregularly shaped gonads were observed in 20.8% of the mullets from the most polluted site, and intersex gonads in 10.3% of those from the estuarine area. No alterations were detected in the fish from the reference site, which also showed distinct stages of gonadal development. Conversely, unclear stages of testicular and ovary development were observed in the fish from the two polluted river sites. The results of this study suggest that *L. ramada* may represent a sentinel species in environmental risk assessment and support the use of gonadal alterations of this species as a bioindicator for extensive monitoring of pollution in lower stretches of rivers and estuarine areas.

## 1. Introduction

Fish are commonly used as bioindicators in aquatic ecosystems, because they may respond to environmental stresses at the different levels of biological organization, from sub-cellular to community levels [1,2,3]. Surveys have often been carried out by evaluating gross indices, such as the hepato- and gonado-somatic indexes (HSI and GSI, respectively), which can provide information related to the fitness of fish and their reproductive maturity, but also on their energy reserves, possible diseases, and exposure to exogenous stresses [4,5]. More recently, fish sexual disorders have been identified as biomarkers of exposure to chemical contaminants in many aquatic environments around the world [6,7,8]. Thus, the anatomical, histological, and biochemical evaluation of the status of the gonads of wild fish populations has been increasingly used to assess the impact of pollution on aquatic ecosystems [9,10,11,12], also with respect to the possible reflections on human health.

Among the contaminants of human origin, the endocrine disrupting chemicals/compounds (EDCs) have an effect analogous to sex steroids and can cause a wide variety of impairments in fish [13], including reduction in gonad weight and volume, disorders in gonadal maturation, gonadal atresia, and especially an increased occurrence of intersex [9,10,11,12]. Intersex, which is the condition whereby there is the simultaneous occurrence of male and female cells in the same gonad [7,14], have been detected in wild populations of several aquatic animals and interpreted as a signature effect of exposure to EDCs [8,15,16].

Among the EDCs associated with the occurrence of gonadal disorders in fish, an important role seems to be played by the alkylphenols, derived from the degradation of surfactants [17] and commonly detected in the discharge of sewage treatment plants [16,18]. Accordingly, intersex gonads have been observed in several freshwaters fish species inhabiting both lotic and lentic ecosystems exposed to this type of contamination. A high rate of intersex cases and other sexual disorders have been detected in male roach (*Rutilus rutilus*) [16] and gudgeon (*Gobio gobio*) [18] populations exposed to sewage treatment plants discharges in UK rivers [19]. Similar observations have been made in barbels (*Barbus plebejus*) [20] and common carps (*Cyprinus carpio*) [21,22] collected from ecosystems exposed to domestic and industrial wastewaters.

Sexual alterations have also been observed in marine-euryhaline fish living in polluted ecosystems, such as mullets (family *Mugilidae*), as recently reviewed by Ortiz-Zarragoitia *et al.* [23]. Mullets are considered gonochoristic species, and hermaphroditism and gonadal abnormalities appear to be uncommon among individuals living in unpolluted environments [24]. However, several studies have reported the occurrence of gonadal disorders, including the intersex condition, in *Mugil cephalus* [25,26], *Chelon labrosus* [11,12], and *Liza ramada* [27]. Some of them associated the phenomenon with the exposure to chemical contaminants, in particular EDCs [11,12,23].

The family *Mugilidae* contains many species widely distributed all around the world and living in numerous habitats, including river, estuarine and coastal waters [23,28]. Mullets are bottom dwellers, tolerate polluted environments, and appear to be sensitive organisms to EDCs [11,12,23,25,26]. Therefore, they have been proposed as sentinel organisms of environmental pollution [12,23,26,28,29].

Within Mediterranean mullets, the thinlip grey mullet *Liza ramada* (Risso, 1827) is one of the most abundant species, frequent in riverine ecosystems due to its high adaptability to low salinities and water pollution [30]. Fry migrate in schools from sea into estuaries, lower reaches of the rivers, and coastal lagoons or lakes, where they find more favorable trophic conditions, before getting back to the sea for the spawning phase [23,31]. As for other mullets [23], migrating juveniles (young-of-the-year) remain in inland waters during growing and sexual differentiation and maturation phases. Complete maturity is reached at 2–3 years in males and at 3–4 years in females, when fish total length is between 25 and 32 cm [31,32,33]. The thinlip grey mullet is the most common species in the estuarine zones and the lower stretches of many large Mediterranean rivers. In particular, a consistent population of thinlip grey mullet is present in the Tiber River (Central Italy), between the river mouth (Tyrrhenian Sea) and the stretch downstream the city of Rome, where a large wastewater treatment plant effluent is located. Periodic monitoring of the water course carried out by the Regional Environmental Protection Agency through chemical, microbiological and biological indexes, accounted for a status of severe pollution for this river stretch [34]. In particular, the presence of high concentrations of polycyclic aromatic hydrocarbons and nonylphenols in water and sediments has been reported [35]. Accordingly, studies conducted in this river stretch showed acute toxicity of water samples to *Daphnia* [36], the presence of persistent organic pollutants [37] and toxic trace elements [38] in the tissues of eels (*Anguilla anguilla*), and alteration of the benthic macroinvertebrates community [36]. Recently, we have also observed a high rate of intersex gonads among the thinlip grey mullet population in the low stretch of the Tiber River [27]. This preliminary finding was pivotal to the present work, which was aimed at evaluating the use of *L. ramada* as a sentinel species for river biomonitoring and ecosystem health assessment. This goal was pursued by comparing the reproductive status and the prevalence of gonadal alterations in mullets belonging to the same population but collected from sites characterized by different levels of pollution.

## 2. Experimental Section

### 2.1. Description of the Study Area

The study area was located in the lower stretch of the Tiber River, downstream of the effluent discharges of a large sewage treatment plant of the city of Rome (mean processed annual flow 8.21 m^3^∙s^−1^). Here, two sampling sites were considered: site A (Lat: 41.812329° N; Lon: 12.419330° E), located immediately downstream of the treatment plant, at a distance of approximately 22.5 km from the sea, and site B, placed in the estuarine area (Lat: 41.752351° N; Lon: 12.275301° E), at a distance of 0.4 km from the sea. Both sites are characterized by high levels of contamination, mainly due to the sewage treatment plant discharge in site A and to agricultural runoff in site B. No impassable barriers are present between site A and B, but the distance between the two sites makes significant population exchanges unlikely. A third site (site C), acting as reference site, was an uncontaminated freshwater semi-natural pond located within an artificial wetland of the Laboratory of Experimental Ecology and Aquaculture (LEEA) (Lat: 41.851442° N; Lon: 12.630322° E) of the University of Rome “Tor Vergata”. The surface area of this pond is 2.200 m^2^, with an average depth of about 1.5 m. The pond is supplied by an artesian well providing unpolluted water (L. Mancini, personal communication). Thinlip grey mullets stocked in the pond were collected as fingerlings at the mouth of the Tiber River in 2004 and therefore belonged to the same population as those collected from the other two sites. To simulate a wild-like ecological condition, fingerlings were stocked at a low density, and fish feeding requirement relied just upon the natural feeding resources of the pond.

### 2.2. Sample Collection

Three sampling campaigns were carried out in summer 2010, winter 2010/2011, and spring 2011. The sampling periods corresponded respectively to the periods of gonad maturation, spawning, and post-spawning of *L. ramada*.

At each sampling campaign, physical and chemical parameters of the water (dissolved oxygen, temperature, and pH) were measured *in situ* using a WTW MultiLine P4 probe (WTW GmbH, Weilheim, Germany).

The procedures used for fish catching were carried out in agreement with the relevant legislation (CEN EN 14011/2003—Water quality). Fish sampling was authorized (n. 526425) by the competent department of the Lazio region. Treatments and experimental handling of animals were carried out according to the Italian regulations (Dlgs. 116/92) and authorized by the Institutional Animal Care and Use Committee of the University of Rome “Tor Vergata”, responsible for reviewing all protocols involving live animals and ensuring compliance with national regulations.

Thinlip grey mullets were collected by gill nets with a mesh size of 40 mm. Adult fish with an approximate total length (TL) greater than 30 cm were immediately euthanized using an overdose of eugenol (330 ppm, Sigma-AldrichCo. LLC, Milano, Italy).

### 2.3. Sample Observation

TL (mm) and body weight (g) were recorded on each fish. A first anatomic examination was carried out for sex determination and gross alteration of shape/color/dimensions of gonad. Liver and gonads from each fish were dissected out and weighted, in order to calculate the gonado-somatic (GSI, gonad weight × 100/total body weight—gonadal weight) and hepato-somatic (HSI, liver weight × 100/total body weight—liver weight) indices [16].

Cross-sections (8–10 mm) of the central portion of each left gonad (or of the gonads which exhibited anomalies at the external examination) were fixed in Bouin’s fluid for 24–48 h, dehydrated through a graded alcohol series, cleared with xylene and finally embedded in paraffin wax. Tissue sections (5 μm thick) were stained with hematoxylin and eosin, and five histological slides from each left gonad were examined under a light microscope (Wild Leitz GMBH, Wetzlar, Germany).

Gonadal development stages were determined following the classification described by Jafri [39]. Oocyte maturation was divided into four different stages, according to the cell size and morphology, and to the extent of yolk accumulated: primary oocytes (stage 1), pre-vitellogenic oocytes (stage 2), secondary oocytes (stage 3), and degenerated oocytes (stage 4). Four spermatogenetic stages were differentiated according to the morphology and the size of the cells: spermatogonia (stage 1), spermatocytes (stage 2), spermatids (stage 3) and spermatozoids (stage 4). Since several stages may coexist in a given gonad, the sexual maturity of fish was defined according to the most advanced stage occurring inside the gonad. The occurrence of gonadal anomalies, based on gross morphology and on histology, was assessed according to Hecker *et al.* [7], as reported in Table 1.

**Table 1:** Assessment and classification of gonadal anomalies in *Liza ramada*, according to Hecker *et al.* [7].

Title	Term	Diagnostic Description
Gross morphology level	Degeneration of gonadal tissue (Castration according to Hecker *et al.* [7])	Removal of the gonads or their destruction as by external influence resulting in a non fertile organism
	Segmented gonads	Gonads are segmented into discrete subunits with obvious gonadal tissue separated by thin pieces of connective or non gonadal tissue
Histology level	Intersex (Mixed gonadal tissue)	Testicular and ovarian tissues occur in the same individual; phenotypic sex is unclear
	Intersex (Testicular oocytes)	Oocytes present in the testes regardless of maturation stage

### 2.4. Statistics

All metrics were reported as mean ± standard deviation. PAST Software version 2.13 [40] was used for statistical analyses. The normal distribution of data was verified using the Kolmogorov–Smirnov test. Data were analyzed by One-Way Analysis of Variance, and significant differences (*p* < 0.05) resulting from the test were reanalyzed by the Least Significant Difference, to determine which individual groups were significantly different from the control group.

## 3. Results

### 3.1. Physical and Chemical Analyses of the Water at the Sampling Sites

Water physical and chemical features at each sampling site are shown in Table 2.

**Table 2:** Physical and chemical water parameters at the sampling sites.

Sampling Site	Sampling Season	T °C	pH	O_2_ %
**A:** Tiber River, downstream of the sewage treatment plant	*Summer 2010*	21	7.7	34.7
	*Winter 2010/2011*	10	7.8	84.8
	*Spring 2011*	18	7.8	52.5
**B:** Tiber River, estuarine area	*Summer 2010*	22.5	7.7	41.1
	*Winter 2010/2011*	13	7.7	81.3
	*Spring 2011*	17	7.6	70.6
**C:** LEEA pond, uncontaminated reference site	*Summer 2010*	24	7.9	84.9
	*Winter 2010/2011*	15	8.1	97.8
	*Spring 2011*	20	7.8	82.4

### 3.2. Fish Analysis

A total of 206 thinlip grey mullets were collected and examined. Sex, mean TL, and mean weight of the fish are reported in Table 3, according to the sampling sites. TL and weight of the mullets from the two river sites were higher than those of fish from the reference site, with statistically significant differences for fish collected in summer and winter (data not shown; one-way ANOVA, *p* < 0.001).

**Table 3:** Characteristics of the thinlip grey mullets collected from the Tiber river sites (A and B) and the LEEA pond (site C). M: Male; F: female; GA: fish with gonadal anomalies detected at gross anatomical observation (irregularly shaped/colored gonads). For each parameter, statistically significant differences between the sites, for *p* < 0.05, are shown with asterisks (*).

Sampling Site	N. of Fish Examined	Sex of Fish Examined			Fish Size (M-F-GA)	
		M	F	GA	TL (cm)	Weight (g)
**A**	101	58	36	7	39.4 ± 11.4	702 ± 280
**B**	58	29	26	3	37.7 ± 13.4	671 ± 311
**C**	47	19	28	0	31 ± 10.5 *	321 ± 124 *
**Total**	206	106	90	10		

**Figure 1:**
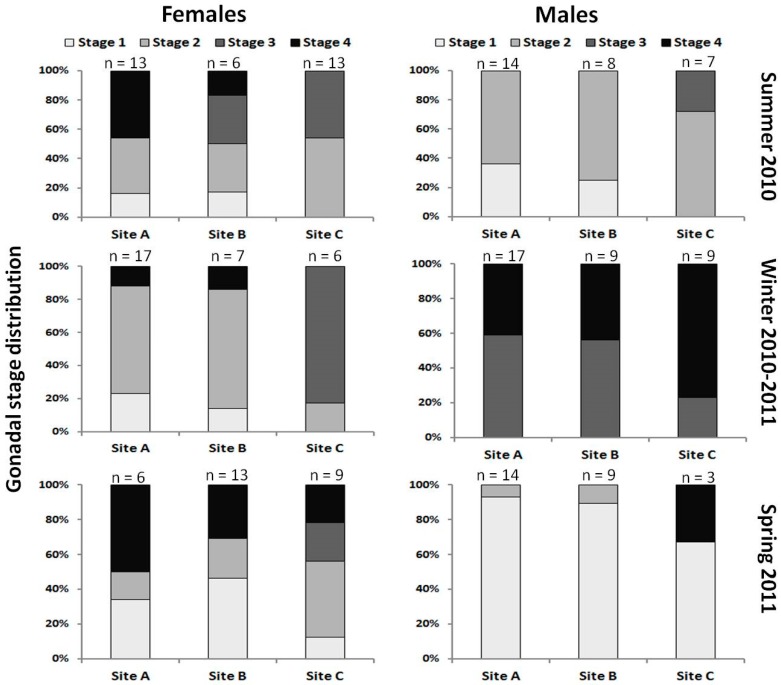
Distribution of the gonadal development stages in female and male mullets sampled in Summer 2010, Winter 2010–2011 and Spring 2011 from the Tiber river site A and B, and from the uncontaminated reference site C. In female mullets: stage 1 = primary oocytes, stage 2 = pre-vitellogenic oocytes, stage 3 = secondary oocytes and stage 4 = degenerated oocytes. In male mullets: stage 1 = spermatogonia, stage 2 = spermatocytes, stage 3 = spermatids and stage 4 = spermatozoids.

Well defined stages of gonadal development were observed in both male and females fish from the unpolluted reference site C, according to the sampling season and the corresponding stage of the reproductive cycle (Figure 1). Conversely, only poorly defined stages of testicular and ovary development were observed in the fish sampled at the river sites A and B, irrespective of sampling season. The distribution of gonadal development stages in female and male mullets is reported in Figure 1, according to the sampling site and season. In winter, in correspondence with the reproductive period of the species, the gonads of male fish from sites A and B showed approximately 60% of spermatids (stage 3) and 40% of spermatozoids (stage 4) as the most advanced development stages, against approximately 20% of spermatids and 80% of spermatozoids observed in the fish from the reference site C. In spring, fish testes were empty, with an immature virgin stage characterized by the presence of spermatogonia and few spermatocytes. One individual from site C was in the spent stage and showed residual spermatozoids. In summer, the testes of fish from sites A and B still showed spermatogonia (approximately 35% of fish from site A and 25% from site B) and spermatocytes (approximately 65% of fish from site A and 75% from site B), while the presence of spermatids (approximately 30%) was recorded only in fish from site C.

As far as the female gonads were concerned, in winter most of the fish from site C (83%) showed secondary stage oocytes, while fish from sites A and B showed only the first two gonad development stages and ovary atresia. In spring, extended ovary atresia was observed in all the mullets from sites A and B, which showed only oogonia and disperse perinuclear oocytes as the most advanced development stages. A few secondary oocytes were observed in 22% of the fish from site C.

In summer, ovary atresia was observed again in 46% of the fish from site A, while oocytes at different maturation stages were present in the females from the less polluted site B and from the reference site C. In particular, fish from site C presented only pre-vitellogenic (54%) and secondary oocytes (46%), while fish from site B showed primary oocytes (approximately 33%) and pre-vitellogenic and secondary oocytes at similar rates.

**Figure 2:**
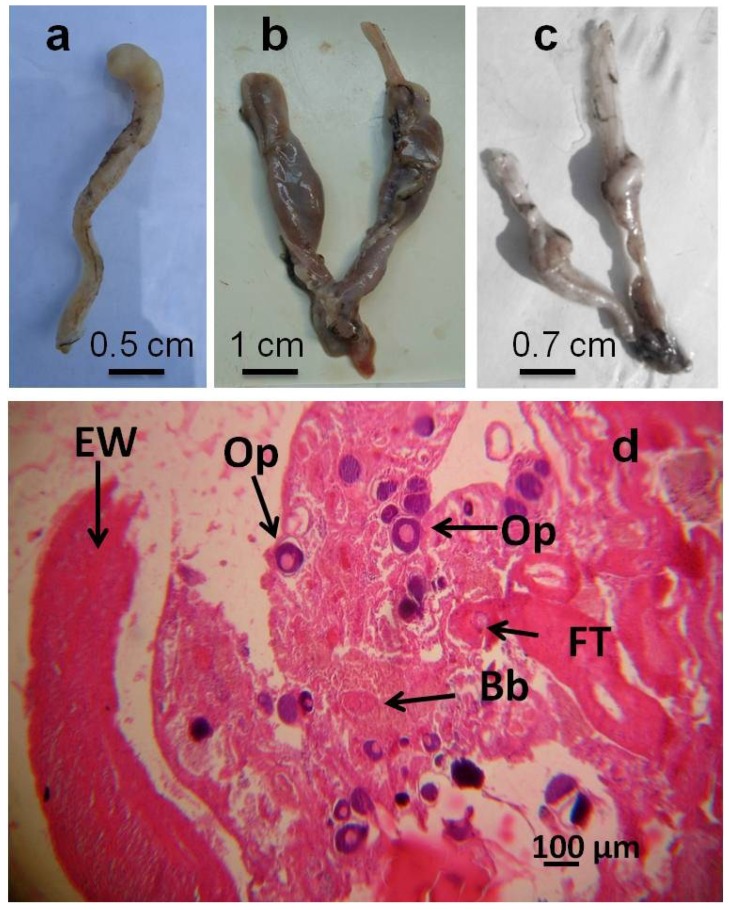
(**a**–**c**): example of irregularly shaped ovaries observed in mullets from site A of the Tiber river. Note the shape and color differences. (d): histological section of one of these ovaries, showing the thicked external wall (EW), the presence of primary oocytes (Op), fibrotic tissue (FT), and degenerative structures known as brown bodies (Bb), which represent a defined stage of the ovarian atretic process and indicate that gonads are in phase of regression.

According to the classification of Heker *et al.* [7], gonadal anomalies were observed in 27 (17%) of the mullets collected from the Tiber sites A and B, but not in the fish from the uncontaminated reference site C. In 10 individuals the anomalies were detectable from gross examination (GA in Table 3). Three of these fishes (all collected at site A) showed irregularly shaped ovaries (Figure 2a–c), which appeared fibrotic and enveloped by a thick capsule hard to cut. Color ranged from straw pale to dark brown and black (Figure 2a–c). Further, they resulted characterized by the lack of the internal cavity and the scarcity of gonadal cells (Figure 2d).

**Figure 3:**
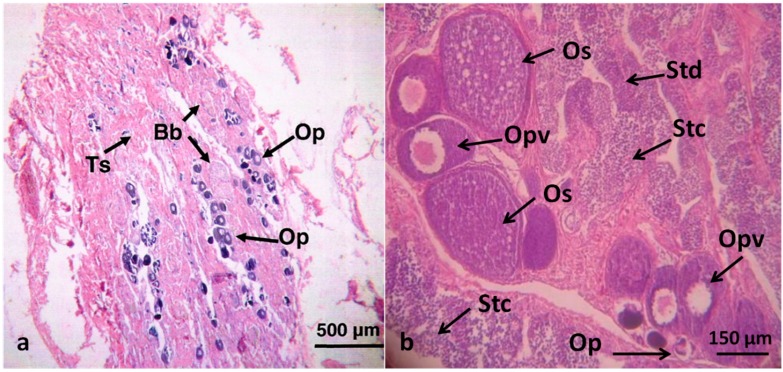
Micro-photographs of a typical testicular oocyte (intersex gonad), showing a mixture of ovarian and testicular elements. Ts: seminiferous tubules; Stc: spermatocytes; Std: spermatids; Sp: spermatozoids; Op: primary, Opv: previtellogenic and Os: secondary oocytes; Bb brown bodies.

All the other gonadal anomalies externally detectable, four from fish sampled in the site A and three from site B, (Table 3) consisted of segmented gonads. These cases were characterized by the concomitant presence of testicular and ovarian tissues in the same gonad but compartmentalized in a left–right or rostral–caudal separation of the ovarian and testicular components. The gonad morphology of these samples has been previously described [27]. The other 17 intersex gonads were cases of testicular oocytes [7], with oocytes at different stages of development (primary, previtellogenic and secondary stages), randomly dispersed in the testicular tissue (Figure 3) and they were identified histologically.

The overall intersex prevalence was not significantly different between the two polluted Tiber river sites (17.8% at Site A *vs.* 10.3%, at Site B, *p* = 0.09, Table 4). The highest intersex rate was observed in winter (A+B: 21.5%), in correspondence of the reproductive period of the species. Intersex gonads were also observed in 17.6% of the fish collected in summer, the period of gonad development, but not in spring, during the post-spawning phase (Table 4).

**Table 4:** Prevalence of gonadal anomalies in thinlip grey mullets collected from the Tiber river sites A and B.

Sampling Site	Sampling Season	Number (%) of Fish with:		
		Examined	Irregularly Shaped Gonads	Intersex Gonads
A	Summer 2010	35	2 (5.7)	7 (20.0)
	Winter 2010/2011	45	0	11 (24.4)
	Spring 2011	21	1 (4.8)	0
	*Total*	*101*	*3 (2.9)*	*18 (17.8)*
B	Summer 2010	16	0	2 (12.5)
	Winter 2010/2011	20	0	4 (20.0)
	Spring 2011	22	0	0
	***Total***	*58*	*0*	*6 (10.3)*
*TOTAL*		*159*	*3 (1.9)*	*24 (15.1)*

### 3.3. Gross Indices

The HSI and GSI values are reported in Table 5, according to gender, presence of gonadal anomalies, sampling site, and season. Among the gross indices, HSI values of fish from the unpolluted site C were lower than those of fish from both river sites A and B, regardless of the gender, with significant differences for fish collected in summer and winter (One-way ANOVA, *p* < 0.05).

Conversely, GSI values were higher in fish from site C than in those from sites A and B. In winter, differences were statistically significant for both males and females (One-way ANOVA, *p* < 0.001 and *p* < 0.05 respectively), while in summer only for females (*p* < 0.05). In spring, females from site A showed GSI values significantly lower than those from sites B and C (*p* < 0.05). The GSI values of both males and females from the uncontaminated site C peaked in winter, in correspondence with the reproductive season, and reached the lowest levels in spring in correspondence with the quiescent phase (One-way ANOVA, *p* < 0.05). On the contrary, no significant seasonal variations were observed for the GSI of fish collected at the polluted sites A and B.

**Table 5:** Gross indices (HSI and GSI) in thinlip grey mullets collected from the Tiber river sites (A and B) and the LEEA pond (site C), according to the sampling season. Data (mean ± SD) are presented separately for male (M), female (F), and fish with gross gonadal anomalies (GA) detected at gross examination. *n* = number of fish examined. For each parameter, asterisks (*) denote statistically significant differences between the sites, for *p* < 0.05.

*Sampling Season*			Gross Indices in Fish Collected at:					
			Site A	*n*	Site B	*n*	Site C	*n*
***Summer 2010***	HIS (%)	M	1.8 ± 0.4	14	2.9 ± 1.6 *	8	0.8 ± 0.2	7
		F	1.7 ± 0.3	13	1.6 ± 0.6	6	0.8 ± 0.2 *	13
		GA	2.0 ± 0.3	7	2.2 ± 0.9	2	-	-
	GSI (%)	M	0.1 ± 0.1	14	0.4 ± 0.5	10	0.4 ± 0.7	7
		F	0.2 ± 0.2	13	0.1 ± 0.05	6	0.5 ± 0.3 *	13
		GA	0.1 ± 0.1	7	0.1 ± 0.1	2	-	-
***Winter 2010/2011***	HIS (%)	M	1.7 ± 0.4	17	1.6 ± 0.3	9	0.7 ± 0.2 *	9
		F	1.7 ± 0.3	17	1.6 ± 0.2	7	1 ± 0.4 *	6
		GA	1.9 ± 0.3	11	1.7 ± 0.3	4	-	-
	GSI (%)	M	0.2 ± 0.1	17	0.1 ± 0.1	9	1.1 ± 0.5 *	8
		F	0.3 ± 0.3	17	0.1 ± 0.1	7	2.8 ± 4.6 *	3
		GA	0.2 ± 0.1	11	0.1 ± 0.04	3	-	-
***Spring*** 2011	HIS (%)	M	1.8 ± 0.3	14	1.7 ± 0.5	9	1.4 ± 0.8	3
		F	1.9 ± 0.3	6	1.4 ± 0.2	13	1.5 ± 0.8	9
		GA	2.37	1	-	-	-	-
	GSI (%)	M	0.1 ± 0.05	14	0.1 ± 0.1	9	0. 1 ± 0.02	3
		F	0.2 ± 0.1 *	6	0.4 ± 0.2	13	0. 4 ± 0.05	9
		GA	0.3	1	-	-	-	-

## 4. Discussion

Reduced development and morphological alterations of the gonads, including intersex, have been described in several fish species living in polluted water systems [16,20,22], and have been associated to exposure to ECDs [10] and other contaminants released with civil and industrial discharges [16,18,41]. In this study, we observed the presence of intersex and irregularly shaped gonads only in thinlip grey mullets living in two sites of the same riverine ecosystem, both located downstream the effluent of a large urban sewage treatment plant and characterized by high levels of contamination. Conversely, gonadal anomalies were not observed in fish belonging to the same population but moved and stocked in the unpolluted reference site C, where fish had undergone growth, sexual differentiation, and maturation. This result confirms that the prevalence of natural hermaphroditism in mullets is non-existent [24] or very low [42].

Fish from the reference site were significantly smaller than those from the polluted river sites, but this would not have influenced the detection of gonadal anomalies. The smaller size was likely due to the oligotrophic conditions of the unpolluted site, and not to differences in age. Moreover, fish from the reference site showed better defined stages of gonadal development than those from the river sites A and B, making the identification of gonadal anomalies easier.

Prevalence of intersex gonads comparable to, or higher than, those observed in this study has been reported in other mullet species sampled from polluted environments: 21% in mullets (*Mugil cephalus*) from the Douro estuary, northern Portugal [26], and up to 50% in male thicklip grey mullet (*Chelon labrosus*) populations in the Bay of Biscay, northern Spain [11,12].

In this study, the prevalence of intersex and irregularly shaped gonads was higher in mullets collected from the highly polluted river stretch (site A, 20.8%) than in those from the less polluted estuarine area (site B, 10.3%), although the difference was not significant. A correlation between the frequency of fish with intersex gonads and the amount of urban sewage discharge in the rivers has been previously reported by Jobling *et al.* [16] and could be due to EDCs such as the alkylphenol polyethoxylates [43], that may derive from the degradation of surfactants in sewage treatment plants [16,17,18]. Moreover, the effects of chemical pollution can be exacerbated by situations of chronic environmental hypoxia, which can also affect the endocrine system of fish and cause extensive reproductive disruptions, including ovarian masculinization and alterations of gonadal development and gamete production [44,45]. The two sites on the Tiber river included in this study, in particular site A, are characterized by pollution due to civil and industrial drains [36,38], with the presence of high levels of detergent derivatives such as the alkylphenols [35], and also by low levels of dissolved oxygen. The latter can, especially in summer time, cause prolonged periods of hypoxia that could have contributed to the occurrence of the detected gonadal anomalies, though no cases of ovarian masculinization were observed in this study. In this strongly impacted environment, *L. ramada* population is exposed to contaminants during the sexual differentiation and maturation periods [23,24], that in other fish species represent the most sensitive exposure moments for intersex induction [46,47]. This could explain the high rate of intersex observed in mullets from the river sites against the absence of gonadal disorders in those from the reference site.

Intersex gonads observed in this study included alterations either in the gross morphology or at the histological level. All the histological alterations were cases of testicular oocytes, which represent the most common gonadal anomaly observed among fish and amphibians [7,14,48]. Similar histological pictures have been reported for most of the intersex cases described in *M. cephalus* [26] and *C. labrosus* [11,12].

A marked seasonal pattern in the incidence of intersex in *L. ramada* was observed in the present study. Intersex gonads were observed in winter and summer, that in this species and in these sites correspond to the periods of spawning and gonad development, respectively, but not in spring, during the recrudescence phase. Similar results have been reported by Blazer *et al.* [9], who observed the highest prevalence of intersex in smallmouth bass during the prespawn season and a decrease in its incidence in correspondence of postspawn. Accordingly, Barrett and Munkittrick [49] recommended sampling two to three weeks before the beginning of the spawning season, to maximize sensitivity in the evaluation of the impact of environmental pollution on the reproductive condition of wild fish. However, according to the review by Bahamonde *et al.* [14], the observation of seasonal dependence of intersex has been rarely considered and most studies that reported intersex in wild fish have been conducted in a single season. Our study confirms that the choice of the sampling period is an important issue in investigating the intersex condition in wild fish, and that timing should take into account the reproductive strategies of the species under study. The optimization of the sampling moment would increase the detection of intersex, with less variability in the results of bio-monitoring programs based on the observation of gonadal disorders in fish [14].

Beside the occurrence of intersex, the gonads of both male and female mullets from the Tiber River polluted sites showed poorly defined stages of testicular and ovary development, while the fish collected from the unpolluted reference site C showed the expected gonadal development according to the sampling season. Such a disturbance of the reproductive cycle was reflected by the GSI values of both male and female individuals from the polluted river sites, which were significantly lower than those of the fish from the unpolluted reference site C in winter, in correspondence with the reproductive season of *L. ramada*. This observation is in agreement with other studies that proposed the measurement of GSI as a valuable tool to detect pollution mediated effects on fish gonads [41,50,51] and suggests that GSI can represent a useful marker to evaluate reproductive impairment in mullets living in polluted areas.

The high HSI values observed in the mullets from the polluted sites A and B are more difficult to be interpreted and further studies are needed to evaluate the HSI values of populations of *L. ramada* sampled from different environments. On the other hand, our results are in agreement with previous studies on fish exposed to the discharge of sewage treatment plants [52], which also reported HSI values higher than those of control individuals.

## 5. Conclusions

The results of this study support the possible use of gonadal alterations in thinlip grey mullet in the pre-spawning phase as a bioindicator of environmental pollution, confirming that mullets may represent valuable sentinel organisms [23]. Although further studies are needed to widen the number of investigated populations, *L. ramada*, owing to its ecology and life cycle, appears to be a good candidate for extensive monitoring of pollution in transitional waters, and, more generally, to carry out environmental risk assessments in inland and coastal water ecosystems.
